# The Effects and Adverse Reactions of Different Bowel Cleansers on Gut Microbiota After Colonoscopy

**DOI:** 10.5152/tjg.2025.25150

**Published:** 2025-08-11

**Authors:** Jiabin Hu, Xiongqun Peng, Shuang Cai, Jiaojiao Qiu, Shuxia Wang, Ping Zhu

**Affiliations:** 1 Department of Geriatrics, Chinese PLA General Hospital, The Second Medical Center and National Clinical Research Center for Geriatric Diseases, Beijing, China; 2 Medical School of Chinese PLA, Beijing, China; 3 Department of Health Service, Guard Bureau, Joint Staff Department, Beijing, China; 4 Department of Gastroenterology, The Affiliated Changsha Central Hospital, Hengyang Medical School, University of South China, Hunan, China; 5 Outpatient Department, of the 51st Retired Cadre Rest Home, Beijing, China

**Keywords:** Adverse reactions, bowel cleansers, bowel cleansing, colonoscopy, gut microbiota

## Abstract

Colonoscopy is a pivotal technique for screening and diagnosing colorectal diseases, particularly colorectal polyps and cancers. Early detection of lesions enables early diagnosis and treatment, thereby improving patient prognosis. The adequacy of bowel preparation is directly related to the quality and effectiveness of colonoscopy. However, the bowel cleansing process may disrupt the balance of gut microbiota, subsequently affecting the host’s health status. Currently, there is a relative scarcity of reports on the specific impacts of different types of laxatives on gut microbiota. Therefore, this paper aims to deeply analyze and summarize the effects of various bowel preparations on gut microbiota and their potential adverse reactions following colonoscopy, providing a more comprehensive reference for the selection of bowel preparations in clinical practice.

**Main Points**

- The adequacy of bowel preparation is directly related to the quality and effectiveness of colonoscopy. For instance, inadequate bowel preparation may lead to the omission of certain lesions, particularly early-stage colorectal cancer.
- At present, the commonly used bowel cleansers in clinical practice mainly include polyethylene glycol electrolyte solution, magnesium salt preparations, and sodium salt preparations
- Different bowel cleansing agents not only affect the quantity and structure of intestinal microbiota, but also influence the temporal dynamics of intestinal microbiota.
- Different bowel cleansing agents result in varying clinical adverse reactions and acute microecological changes. Whether the impact of bowel cleansing agents on the quantity and species composition of intestinal microbiota is associated with clinical symptoms or long-term metabolic sequelae remains to be determined.
- During the bowel cleansing process for colonoscopy, the intestinal mucosal barrier and microecosystem are compromised to a certain extent, which can subsequently lead to alterations in the intestinal mucosal immune system. This may be the underlying cause of some clinical symptoms.

## Introduction

To reduce or prevent the occurrence of colon cancer, colonoscopy has been recognized as a necessary item in routine health examinations. Adequate and appropriate bowel preparation is crucial for colonoscopy, but this procedure may cause discomfort, adverse reactions, and even unacceptable physical and mental stress to the individuals undergoing the examination. Previous studies have revealed that bowel preparation may induce dysbiosis of the gut microbiota, leading to significant short-term changes in the gut microbiota structure and potential damage to the host’s health.^1^ To date, the specific effects of different bowel cleansers on the gut microbiota have not been clearly elucidated, and the related comparative studies are rare. What adverse reactions may occur after colonoscopy following the administration of various bowel cleansers? Which is the most preferred cleanser? There is currently no consensus on these issues, which are of great significance to clinical practice, making it necessary to conduct more comprehensive research. In response to the current research background, this paper aimed to provide a review on the effects and adverse reactions of different bowel cleansers on the gut microbiota after colonoscopy.

### Types of Bowel Cleansers

At present, the commonly used bowel cleansers in clinical practice mainly include polyethylene glycol-electrolyte solution (PEG-ELS), magnesium salt preparations, and sodium salt preparations.^2^

### Polyethylene Glycol-Electrolyte Solution

Polyethylene glycol is a polymer compound characterized by good solubility and stability. It can form hydrogen bonds with water, thereby increasing the viscosity and surface tension of water. As an osmotic laxative, high-dose administration (2-3L) of PEG-ELS can effectively stimulate intestinal peristalsis, induce watery diarrhea, and exert the effect of intestinal lavage.

### Magnesium Salt Preparations

Magnesium salt preparations mainly include magnesium sulfate and magnesium citrate. Magnesium sulfate has been a widely used osmotic laxative in clinical practice for a long time. After oral administration, its absorption in the body is limited, while unabsorbed magnesium sulfate can produce a hyperosmotic effect, thereby retaining water in the gastrointestinal tract to stimulate intestinal peristalsis. This will lead to defecation reflex to achieve the goal of bowel cleansing. Magnesium citrate has a similar mechanism of action to magnesium sulfate and is more commonly used in certain countries.

### Sodium Salt Preparations

Sodium salt preparations mainly include sodium phosphate (NaP) and sodium sulfate, which are both osmotic laxatives like magnesium salts. As sodium salt preparations do not need to be taken in large doses, they are well tolerated and widely used in bowel preparation for colonoscopy. However, excessive oral administration of sodium salts carries the risk of inducing electrolyte imbalance.

### Mannitol

Mannitol is a highly osmotic and strong dehydrating agent. After oral administration, it will maintain the intestine in a highly osmotic state, which can accelerate the transfer of body fluids to the intestinal cavity, stimulate the intestinal wall, and accelerate intestinal peristalsis, thereby promoting fecal excretion to achieve the goal of bowel cleansing.

### Senna Leaf

Senna leaf is a commonly used irritant laxative, mainly used in clinical practice for the purpose of relieving diarrhea. Its laxative mechanism is mainly activated by the reaction of sennoside A and sennoside B with the stomach and small intestine. The effective components in senna leaf can be decomposed in the liver and enter the bloodstream to induce excitation of the pelvic ganglia, leading to intestinal dysfunction and diarrhea. In comparison, the effect of sennoside A is stronger_._^3^

### Lactulose

Lactulose is composed of fructose and galactose. Due to the osmotic effect of disaccharides, lactulose is capable of promoting excretion by maintaining a highly osmotic state of water and electrolytes in the intestinal cavity. However, due to a relatively mild laxative effect, lactulose is seldom used alone for bowel cleansing in clinical practice. For patients with chronic constipation, lactulose can be used as an adjuvant a few days before bowel preparation in order to improve bowel movements.

### Effects of Different Bowel Cleansers on Gut Microbiota

There are approximately 24 genera and 150-400 species of bacteria in a normal adult’s gut, with a total population of up to 10^14^.^4^ The gut microbiota can be generally divided into 3 categories: 1) beneficial bacteria, accounting for about 99% to the total, including anaerobic gram-positive bacteria such as *Lactobacillus*, *Lactobacillus*, and *Bifidobacterium*; 2) conditional pathogenic bacteria, i.e., non-dominant bacterial groups, such as *Enterococcus* and *Escherichia coli*; 3) pathogenic bacteria, which may lead to diseases when the gut microbiota is dysregulated. The gut microbiota of the vast majority of people is mainly composed of Firmicutes and Bacteroidetes, followed by Proteobacteria and Actinobacteria.^5^ So far, there has been little research on the impact of bowel preparation on the gut microbiota, especially on how the composition of gut microbiota is affected by colonic lavage and how colonic lavage affects human health.

### Effects of Different Bowel Cleansers on the Abundance and Structure of Gut Microbiota

Jalanka et al^6^ found that the compound PEG electrolyte powder, a laxative drug, could quickly cause significant changes in the gut microbiota, with 22% of the participants losing the subject-specificity of their gut microbiota. The authors reported that the number of bacteria in the samples collected immediately after bowel preparation was 34.7-fold lower than that in the baseline fecal samples, and the number of methanogenic archaea produced per gram of feces was significantly reduced by 20-fold. It is noteworthy that above changes returned basically to baseline levels at the 14-day and 28-day follow-ups, respectively. In addition, Jalanka et al^6^ further revealed that the gut microbiota immediately after PEG bowel preparation showed significant differences compared to baseline samples, even at the class or family level. For example, the bacteria related to brominated *Ruminococcus* and Sepal *Ruminococcus* were reduced by 2.5-fold and 2.3-fold, respectively. On the other hand, the members of Proteobacteria and *Clostridium* cluster IV were increased during lavage, with the most significant increases observed in long-chain Dorrella and active *Ruminococcus* (increased by 2.1-fold and 2.7-fold, respectively). Several Proteobacteria, including Sartre and *Serratia*, were also increased by about 2-fold after bowel preparation. In addition, there was a significant change in the ratio between gram-positive and gram-negative bacteria, which was increased from 5.3 (SD = 4.8) at baseline to 9.2 (SD = 7.5) at the first follow-up 14 days after lavage and then returned basically to the baseline level at the 28-day follow-up. The use of PEG for bowel preparation can cause significant disturbance to the gut microbiota, which is attributed to multiple factors including the flushing of the gut microbiota by PEG-ELS, the entry of air during bowel preparation that disrupts the anaerobic environment on which the microbiota relies for survival, and the acceleration of intestinal peristalsis caused by chemical stimulation.^7,^^8^Partial *Clostridium* clusters IV and XIVa, including bacteria associated with *Faecalibacterium prausnitzii*, also decreased during the lavage procedure,^6^ However, the abundance of *Akkermansia* showed no significant change during lavage with PEG.^9^ In terms of microbial abundance, patients after PEG bowel preparation exhibited a decrease in Firmicutes and Bacteroidetes but an increase in Proteobacteria at the phylum level, as well as a decrease in *Lactobacillus*, *Clostridium*, and *Ruminococcus* but an increase in *Haemophilus* and *Neisseria longiformis* at the genus level, while the microbial diversity showed significant changes in both α diversity and β diversity.^7^^,^^10^ Another study highlighted that the largest discrepancy in fecal microbiota before and after undergoing colonoscopy with PEG was the differences in the abundance of Bacteroidetes and Firmicutes genera.^11^ Lorenzo Drago^8^ evaluated the changes in the composition of gut microbiota immediately after PEG bowel preparation and at the 1-month follow-up by performing 16S rDNA ion flow analysis on the patients’ fecal samples. The results showed that at the phylum level, the abundance of Firmicutes was significantly decreased while the abundance of Proteobacteria was significantly increased (both *P* < .05) immediately after colonoscopy, and the gut microbiota returned basically to the baseline level at the 1-month follow-up; at the class level, the abundance of γ-Proteobacteria was significantly increased immediately after colonoscopy, while at 1-month follow-up, the abundance of γ-Proteobacteria was reduced by 3-fold and the abundance of α-Proteobacteria phylum was reduced by 2.5-fold compared to baseline levels; at the family level, the abundance of *Lactobacilli* significantly was decreased while the abundance of *Enterobacteriaceae* was significantly increased immediately after colonoscopy, and at the 1-month follow-up, the abundance of these families was significantly decreased compared to baseline levels; in addition, the abundance of streptococci was increased by 4.0-fold at the 1-month follow-up. In another study focusing on NaP as a bowel cleansing agent,^12^ the authors compared the changes in gut microbiota at 3 time points (i.e., before bowel preparation or baseline, 7 days after colonoscopy, and 28 days after colonoscopy) and calculated the correlation coefficients between the abundance of each microbiota type and the Shannon diversity index. It was found that the abundance of microbiota had basically returned to the baseline level at 28 days after colonoscopy. More specifically, at the phylum level, the percentages of Bacteroidetes, Firmicutes, and Proteobacteria exhibited slight changes across the 3 time points: samples collected on baseline and at 7 days after colonoscopy showed a negative Spearman correlation between Bacteroidetes and Firmicutes; samples collected at 28 days after colonoscopy showed a weakly negative Spearman correlation between Bacteroidetes and Firmicutes without statistical significance. Shaw et al^13^ carried out a study in which participants underwent bowel preparation following a protocol primarily consisting of sodium picosulfate, magnesium citrate, and senna leaf. Through comparative analysis of the microbiota in fecal samples before bowel preparation and after colonoscopy (with a median postoperative duration of 55 days), it was reported that the overall diversity and composition of the gut microbiota basically remained stable, except for a significant increase in the Christensonian family.

### The Temporal Characteristics of the Effects of Different Bowel Cleansers on Gut Microbiota

The temporal characteristics of the effects of different bowel cleansers on gut microbiota differ significantly, but there are some regularities. For example, when examining fecal and colon mucosal samples after bowel preparation, some researchers^14^^,^ ^15^ reported that the bacterial load and α diversity decreased significantly, but the variations seemed to be able to quickly recover within 14 days (though the exact time of recovery has not yet been fully clarified). Jalanka et al^6^ examined the composition of gut microbiota in 23 healthy volunteers who underwent bowel preparation using PEG at 4 time points (i.e., before and immediately after bowel preparation, 14 days after bowel preparation, and 28 days after bowel preparation). It was found that the composition of gut microbiota was altered immediately after bowel preparation, with 22% of the participants losing the subject specificity of their gut microbiota, but the subject specificity as well as the total bacterial count returned basically to baseline levels at the 14-day follow-up. More specifically, the authors divided the participants into a single-dose group and a double-dose group, both of which exhibited significant differences in the gut microbiota immediately after bowel preparation compared to the baseline level. Although the bacterial abundance and microbiota composition recovered within 14 days, the recovery rate showed a significant correlation with the dose protocol: compared to the double-dose group (2 L PEG-ELS divided into 2 doses, with an interval of 12 hours), the single-dose group (2 L PEG-ELS taken at once) exhibited a more severe impact on the gut microbiota and a lower recovery rate. It can be concluded that the double-dose protocol has a less significant impact on the gut microbiota than the single-dose protocol and may be adopted as the preferred method for clinical practice. However, there are also studies suggesting that patients who underwent colonoscopy using PEG presented with less changes in the composition of gut microbiota compared to those who underwent colorectal surgery, and would return to baseline 10 days after examination.^16^ Moreover, there is evidence^8^ indicating that bowel preparation with PEG might have a long-lasting (at least 1 month) impact on the composition and homeostasis of gut microbiota in normal individuals, as the microbiota did not fully recover to the baseline level, especially with a significant decrease in the abundance of *Lactobacilli* (protective bacteria). Another research team^12^ investigated the changes in gut microbiota in overweight adult males after bowel preparation with NaP based on fecal samples collected at 3 time points (before bowel preparation or baseline, 7 days after bowel preparation, and 28 days after bowel preparation). It was found that the relative abundance of the main bacteria (*Prevotella* and Bacteroidetes) remained almost unchanged at the 28-day follow-up compared to the baseline level. From previous studies, it can be inferred that mannitol and PEG have a faster impact on the gut microbiota and can cause more drastic changes in the microbiota. In comparison, the changes in gut microbiota caused by sodium sulfate after bowel preparation are milder and can recover more quickly.

Although only a few studies have examined the short-term effects of bowel preparation on the gut microbiota and most of these studies are based on small sample sizes, it can be concluded that the flushing effect of bowel cleansers can directly affect the gut microbiota, but the specific manifestations may differ among individuals. Overall, previous studies mostly focused on the specific effect of a certain bowel cleanser on the gut microbiota, with a lack of thorough analysis on the consistent changes and differences in the effects exerted by different bowel cleansers. In many cases, due to the absence of detailed descriptions of microbial recovery rates, the microbial changes observed after bowel preparation were inconsistent. Based on the current research findings, the uncertainties about the effects of different bowel cleansers on the gut microbiota can be mainly attributed to 2 reasons, i.e., small sample size (including both healthy subjects and patients) and lack of in-depth analysis, which often resulted in conflicting data. Therefore, in the further research of gut microbiota, it is necessary to fully consider the representativeness of the samples and to ensure a sufficient sample size.

### Adverse Reactions Caused by Different Bowel Cleansers

The clinical adverse reactions and acute microecological changes caused by different bowel cleansers during colonoscopy may vary. It is currently unclear whether the effects of bowel cleansers on the abundance and composition of gut microbiota are related to the clinical symptoms or long-term metabolic sequelae. The intestinal mucosa is protected by a mucous barrier that contains a large number of symbiotic bacteria, which can prevent pathogens and other toxic substances from reaching the epithelial surface. During colonoscopy, the intestinal mucosal barrier and the microbiota are both subjected to damage to a certain extent, which can lead to changes in the intestinal mucosal immune system, thereby causing a range of clinical symptoms.

### Polyethylene Glycol-Electrolyte Solution

A disadvantage of PEG is that it has a strong odor and needs to be taken orally with a large amount of water. When a large amount of lavage solution is quickly passing through the gastrointestinal tract, it may cause a certain impact and damage to the intestinal mucosal barrier and the microecology. For young children, the elderly, and some special patients with dysbiosis (e.g., inflammatory bowel disease and irritable bowel syndrome), PEG may even exacerbate the symptoms of intestinal discomfort.^17^ The common adverse reactions indicated in PEG instructions include abdominal pain, diarrhea, nausea and vomiting, fecal incontinence, electrolyte metabolism disorders (e.g., hyponatremia, hypokalemia), etc. In addition, the elderly, males, and individuals who are taking non-steroidal anti-inflammatory drugs or vitamin C are exposed to an increased risk of kidney injury following bowel preparation with PEG.^18^ It is worth noting that an earlier study^19^ reported a case of epilepsy possibly caused by hyponatremia during the use of PEG for bowel preparation, which deserves serious attention. In addition, there are also reports of systemic allergic reactions after taking PEG, such as urticaria and lymphedema.^20^ According to statistics, 5%-15% of patients are unable to complete bowel preparation with oral PEG due to a large amount of liquid to be taken or a poor taste. It has been shown that adjusting the taste of PEG with sports drink can significantly improve the patients’ tolerance, while controlling the medication speed, massaging the abdomen, and increasing physical exercises during medication can help reduce the risk of vomiting and bloating, increase the proportion of patients who are willing to use PEG again, and improve the quality of bowel preparation.^21^ Moreover, a meta-analysis revealed that multiple-dose protocols could provide better quality of bowel preparation compared to the single-dose protocol, and could increase the proportion of patients who are willing to retake bowel preparation.^22^

### Sodium Salt Preparations

Mirabilite, a traditional Chinese medicine, is mainly composed of sodium sulfate and has been used for bowel preparation for many years in China. Studies have shown that oral administration of NaP solution has similar bowel preparation effects to oral administration of 4 L PEG-ELS, and the patients have better compliance, with fewer gastrointestinal adverse reactions such as nausea, vomiting, and bloating.^23^^,^ ^24^ However, because NaP preparations are hypertonic solutions, they may induce epilepsy, phosphate nephropathy, and arrhythmia related to electrolyte disorders during the bowel preparation process.^25^^-^^27^ A prospective cohort study followed up on 502 participants aged 40 years and above who underwent colonoscopy with NaP solution.^28^ It was reported that up to 34% of participants experienced mild symptoms that affected their normal activity after colonoscopy, with the most common symptoms being abdominal pain (25%) and bloating (11%). Bowel preparation with NaP may cause severe adverse reactions, including fatal electrolyte disorders^29^ and acute phosphate nephropathy. The former includes hypernatremia, hyperphosphatemia, and hypocalcemia, while the latter can cause deposition of calcium phosphate crystals in the renal tubules, leading to irreversible kidney damage. In 1975, acute kidney injury related to the use of NaP was first reported.^30^ Subsequently, following an increasing number of related cases (e.g., Markowitz et al^26^ reported 21 cases of acute phosphate nephropathy in 2005), the clinical application of NaP solution for bowel preparation was restricted. However, a study focusing on individuals aged 18-55 without underlying kidney disease revealed that NaP solution not only delivered similar or better bowel cleansing effects but also had a lower incidence of bloating or nausea, as well as better tolerance and sleep quality, compared to PEG and magnesium sulfate solutions. Besides, prospective and retrospective studies have shown that although NaP can lead to severe adverse reactions in high-risk patients, its use is generally safe in healthy adults under the age of 55 and with normal kidney function.^14^^,^^31^ Another experimental study compared the use of NaP tablets and PEG-ELS for adult colonoscopy by focusing on the differences in terms of bowel cleansing efficacy, patient compliance, acceptability, satisfaction, safety, and the adenoma detection rate. The results showed that there were no significant differences in bowel cleansing efficacy, patient compliance, overall adverse events, and the adenoma detection rate between the 2 groups, but patients in the NaP group had significantly higher acceptance to the drug taste, better satisfaction, and higher evaluation score than those in the PEG group.^32^ In healthy young (<60 years old) individuals without comorbidities, NaP tablets had similar bowel cleansing efficacy; fewer adverse effects; and higher acceptance, satisfaction, and taste score than PEG-ELS. Moreover, several studies^33^^-^^36^ argued that oral administration of NaP tablets could reduce the burden of bowel preparation to the body, thereby allowing more patients to participate in colonoscopy screening, and had better effects than oral administration of NaP solution or PEG-ELS. However, given the potential risk of NaP, current guidelines do not recommend the routine use of oral NaP for bowel preparation. At present, it is only prescribed for specific situations, especially for patients who cannot tolerate high-dose oral administration of other bowel cleansers.^2^^,^ ^37^

### Magnesium Sulfate

Oral administration of magnesium sulfate produces a bitter and salty taste, which is usually difficult for patients to accept, and compared with PEG, its bowel cleansing effect is weaker, accompanied by significantly more adverse reactions. A study including 314 patients showed that magnesium sulfate had a significantly lower cleansing efficiency and higher incidence of adverse reactions than PEG for bowel preparation.^2^ Because the patients have to drink a large amount of water within a short time, they may easily experience gastrointestinal adverse reactions such as abdominal pain, bloating, nausea, and vomiting during bowel preparation with magnesium sulfate. Besides, due to a high osmotic dehydration effect, magnesium sulfate can lead to a large amount of fluid loss in the body, which can easily cause a decrease in blood volume or electrolyte disorders. For patients with a history of poor kidney function, when a large number of magnesium ions enter the body in a short time, they cannot be easily metabolized and may lead to an increased risk of hypermagnesemia. The aggregation of magnesium ions has been associated with the risks of intestinal mucosal inflammation, ulcers, and dehydration-induced hypermagnesemia, so magnesium sulfate is currently not recommended for patients with inflammatory bowel disease, suspected inflammatory bowel disease, or renal dysfunction.^2^

### Compound Sodium Picosulfate

Compound sodium picosulfate, approved for clinical use in China in 2018, is a composite preparation composed of sodium picosulfate, magnesium oxide, and magnesium citrate. Specifically, sodium picosulfate can increase the frequency and intensity of peristalsis in the colon, while magnesium citrate can retain the fluid in the colon through permeation, thereby achieving the effect of bowel cleansing. Leitao et al^38^ found that sodium picosulfate/magnesium citrate delivered comparable cleansing effects to oral administration of 4L PEG-ELS and had higher patient compliance and a higher polyp detection rate (34.3% vs. 23.3%). A multicenter phase III clinical trial led by Changhai Hospital of Naval Medical University of China, involving 7 organizations,^39^ compared the effectiveness and safety between compound sodium picosulfate (n = 144) and compound PEG electrolyte powder (n = 146) in patients undergoing bowel preparation for colonoscopy. The results showed that the compound sodium picosulfate group had lower incidence of adverse reactions including nausea and vomiting than the PEG group. Another randomized controlled study comparing sodium picosulfate (n = 64) with 2 L PEG (n = 66) showed that the sodium picosulfate group had higher incidence of headache but lower incidence of vomiting than the PEG group, while there were no statistically significant differences in nausea, abdominal pain, and bloating.^40^ However, since magnesium is excreted through the kidneys, compound sodium picosulfate should be avoided for patients with renal insufficiency.

### Mannitol

Mendoza et al^41^ compared the efficacy between mannitol and PEG in 80 patients undergoing colonoscopy, and the results showed that there was no significant difference in the quality of bowel preparation between the 2 groups (satisfaction rate: 87.5% vs. 90%). The incidence of adverse reactions in the mannitol group (30%) was lower than that in the PEG group (42.5%). However, mannitol does have some obvious adverse reactions. For example, short-term oral administration of a large amount of mannitol can lead to dehydration, diuresis, and even electrolyte disorders. Meanwhile, mannitol may also increase the blood glucose level, which is not suitable for diabetic patients.^2^ More seriously, mannitol can produce hydrogen and methane through the fermentation of intestinal microorganisms, which may cause gas explosions during endoscopic high-frequency electrocoagulation treatment.^42^^,^^43^

### Senna Leaf

In a prospective trial^44^ including 120 patients undergoing colonoscopy, the participants were randomly divided into a PEG-ELS combined with senna leaf group (n = 60) and a PEG-ELS alone group (n = 60). It was found that 90% of the participants in the combination treatment group (vs. 66.7% in the PEG-ELS alone group) had no solid fecal residue in their intestines, suggesting that the combination treatment had significantly better quality of bowel preparation than the PEG-ELS alone treatment. In addition, the combination group had a lower intake of fluid. However, senna leaf contains anthraquinone derivatives, and it has a stronger diarrhea effect and irritancy than other laxatives containing anthraquinone. In case of large-dose intake, senna leaf may lead to a series of adverse reactions, such as bloating, abdominal pain, dehydration, and even intestinal mucosal inflammation, neurological toxicity, epilepsy, etc. It is noteworthy that after entering the intestine, the metabolites of senna leaf will cause color changes on the intestinal wall, which may affect observation and increase the risk of missed diagnosis and misdiagnosis. Besides, senna leaf is a traditional Chinese medicine, and it needs to be prescribed according to syndrome differentiation. Last but not least, the adverse reactions of senna leaf are not yet fully clarified. Based on the above analysis, senna leaf is currently less commonly used in clinical practice or only used as a supplement to other bowel cleansers.

### Lactulose

Lactulose is an osmotic laxative, which is a non-absorbent disaccharide that can be artificially synthesized. It has a sweet taste and can retain water and electrolytes in the intestinal cavity, resulting in a high osmotic effect. It has been reported that constipation patients who took low-dose lactulose solution 1-3 days in advance on the basis of routine bowel preparation achieved better bowel cleansing effects.^45^ Another study^46^ reported that compared to oral administration of PEG electrolyte powder alone, the combination of lactulose and PEG powder could significantly increase the frequency of bowel movements in constipation patients, shorten the time to the first bowel movement time after medication, and improve the bowel cleansing effect. Meanwhile, the combination therapy was safe for patients with constipation, while the incidence of adverse reactions was similar between the 2 groups. However, lactulose is seldom used alone for bowel preparation in clinical practice due to its mild laxative effect.

## Conclusions and Prospect

The quality of bowel preparation directly determines the effectiveness of colonoscopy. Unfortunately, there is currently no absolutely ideal bowel cleanser yet. Each commonly used bowel preparation protocol has its own advantages and disadvantages, so it is of great significance to design personalized protocols based on the specific situation of patients. It has been confirmed that the flushing effect of bowel cleansers can quickly cause obvious changes to the gut microbiota, leading to a significant decrease in the diversity and abundance of the gut microbiota in the patients. More specifically, the most common changes in the gut microbiota include a short-term drastic reduction in specialized anaerobic bacteria (e.g., Firmicutes and Bacteroidetes) and short-term increase in facultative anaerobic bacteria (e.g., Proteobacteria). A vast majority of existing studies have shown that these diversity changes can recover to baseline levels without taking any particular intervention within 14-28 days after colonoscopy. From a clinical perspective, a part of patients may experience discomfort symptoms such as nausea, vomiting, bloating, abdominal pain, diarrhea, and constipation within 7 days after bowel preparation, with an incidence rate of around 30%. Most of such symptoms are mild and last for no more than 24 hours. However, both osmotic and irritant laxatives involve certain safety concerns, presenting with relatively serious adverse effects. Compared to mannitol, sodium salt preparations, magnesium salt preparations, and other commonly used bowel cleansers, PEG is characterized by multiple advantages such as good cleansing effect, high safety, and lower incidence of delayed adverse reactions. Moreover, it has a milder impact on the gut microbiota and faster recovery than some other cleansers. Considering that PEG has already been widely used in clinical practice both at home and abroad, it is worth recommending for general purpose of bowel preparation. Nonetheless, for individuals under the age of 55 and without underlying kidney disease, oral administration of NaP tablets may be a better choice, but more research evidence is warranted.

In the future, it is necessary to conduct further research on the specific effects of different bowel cleansers on the gut microbiota and perform in-depth analysis on the regularity and differences in such effects between different cleansers. In summary, to better facilitate clinical decision-making and clinical application, an ideal bowel cleanser should be characterized by a small impact on the gut microbiota, fast recovery, fewer clinical symptoms, and even benefits to the gut microbiota.

## Data Availability

The data that supports the findings of this study are openly available.
